# mTOR and MEK1/2 inhibition differentially modulate tumor growth and the immune microenvironment in syngeneic models of oral cavity cancer

**DOI:** 10.18632/oncotarget.5063

**Published:** 2015-10-22

**Authors:** Harrison Cash, Sujay Shah, Ellen Moore, Andria Caruso, Ravindra Uppaluri, Carter Van Waes, Clint Allen

**Affiliations:** ^1^ Tumor Biology Section, Head and Neck Surgery Branch, National Institutes of Deafness and Other Communication Disorders, National Institutes of Health, Bethesda, MD, USA; ^2^ Department of Otolaryngology-Head and Neck Surgery, Walter Reed National Military Medical Center, Bethesda, MD, USA; ^3^ Department of Otolaryngology-Head and Neck Surgery, Washington University in St. Louis School of Medicine, St. Louis, MO, USA; ^4^ Department of Otolaryngology-Head and Neck Surgery, Johns Hopkins School of Medicine, Baltimore, MD, USA

**Keywords:** head and neck/oral cancers, signal transduction pathways, inflammation and tumor development, animal models of cancer, immunomodulation

## Abstract

We investigated the effects of mTOR and MEK1/2 inhibition on tumor growth and the tumor microenvironment in immunogenic and poorly immunogenic models of murine oral cancer. *In vitro*, rapamycin and PD901 inhibited signaling through expected downstream targets, but only PD901 reduced viability and altered function of MOC cells. Following transplantation of MOC cells into immune-competent mice, effects on both cancer and infiltrating immune cells were characterized following rapamycin and/or PD901 treatment for 21 days. *In vivo*, both rapamycin and PD901 inhibition reduced primary growth of established MOC tumors on treatment. Following withdrawal of PD901, rapid rebound of tumor growth limited survival, whereas durable tumor control was observed following rapamycin treatment in immunogenic MOC1 tumors despite more robust inhibition of oncogenic signaling by PD901. Characterization of the immune microenvironment revealed diminished infiltration and activation of antigen-specific CD8+ T-cells and other immune cells following PD901 but not rapamycin in immunogenic tumors. Subsequent *in vitro* T-cell assays validated robust inhibition of T-cell expansion and activation following MEK inhibition compared to mTOR inhibition. CD8 cell depletion abrogated rapamycin-induced primary tumor growth inhibition in MOC1 mice. These data have critical implications in the design of combination targeted and immune therapies in oral cancer.

## INTRODUCTION

Outcomes of patients diagnosed with advanced head and neck squamous cell carcinoma (HNSCC) remain poor despite advances in locoregional management. Human papillomavirus (HPV)-associated HNSCC, which represents 20% of all HNSCC, demonstrates good responses to standard anti-cancer therapies with disease-specific survival rates of ≥ 80% [1]. Conversely, half or more of all patients diagnosed with advanced-stage carcinogen-associated HNSCC will die from their disease within 5 years. Further, current treatment strategies often functionally disable patients that do respond well to therapy and lead to long-term quality of life impairment [2]. Clearly, more efficacious treatment options are needed for this patient population.

Emerging data from The Cancer Genome Atlas (TCGA) has identified genomic changes associated with the development of HNSCC [3]. Alterations in *EGFR*, *FGFR1/3*, *IGFR*, *PIK3CA* or *RAS* genes are found in 60% of HPV-negative HNSCC and drive direct or cross-talk activation of the phosphoinositide 3-kinase/mammalian target of rapamycin (PI3K/mTOR) and mitogen activated protein kinase kinase/extracellular related signal kinases 1 and 2 (MEK/ERK1/2) pathways [4]. As such, therapeutic inhibition of these pathways with small molecule inhibitors in models of head and neck cancer have been extensively investigated [5–7].

Benefit versus toxicity from therapy targeting the PI3K/mTOR and MEK/ERK1/2 pathways alone or in combination is a concern [8]. Given the important role of these signaling pathways in a number of physiologic systems, targeted therapies can have both desirable, inhibitory effect on cancer cells as well as undesirable effects on other cell types. No where is this more evident than on cells of innate and adaptive immunity, where different targeted therapies may directly suppress a number of different stimulatory and effector functions [9]. The majority of pre-clinical investigation involves the use of xenograft models, which to do not allow the study of how systemic agents affect adaptive immunity activation. Recognition of how different anti-tumor agents affect immune cell function is critical given the interest in combining targeted and immune-activating anti-cancer therapies [10], but poorly studied.

The murine oral cancer (MOC) model is a syngeneic model that allows study of host anti-tumor immunity. Previous work has demonstrated that MOC1 cells, which exhibit a high genomic alteration rate, generate tumors with increased CD8 T-cell infiltration and increased interferon-γ (IFNγ), MHC class I and programmed death ligand 1 (PD-L1) expression compared to MOC2 tumors in immune-competent mice [11]. Similar to MOC1, roughly two-thirds of HNSCC tumors demonstrate a high degree of genomic alterations and increased immunoreactive infiltrates. Conversely, similar to MOC2, human HNSCCs include a subset of *RAS* mutant tumors with low frequency of genetic alterations and limited immunogenicity [3, 12, 13]. The effects of PI3K/mTOR and MAPK pathway targeting agents on anti-tumor immunity are of interest given the demonstrated activity of immune checkpoint inhibitors in HNSCC [12, 14] and the potential for enhanced patient responses with combining these immune-modulators with targeted therapies.

Here, we characterized the anti-tumor and immune effects of rapamycin, an FDA-approved inhibitor of mTOR signaling, and an investigational MEK1/2 inhibitor PD0325901 in these syngeneic MOC1 and 2 murine models of *Ras*-mutant oral cavity cancer. We validated on-target effects of these agents both *in vitro* and *in vivo* and the ability of both drugs to suppress primary tumor growth while on-treatment, yet demonstrated that MEK inhibition alone consistently resulted in measurable altered MOC cell viability and function with little effect following mTOR inhibition. Paradoxically, mTOR but not MEK inhibition resulted in durable tumor control following cessation of therapy in immunogenic MOC1 but not poorly immunogenic MOC2 tumors. We demonstrated that this differential response is not due to enhanced tumor cell-specific effects of *in vivo* mTOR inhibition but rather due to preservation of antigen-specific CD8 T-cell responses that are suppressed following MEK inhibition. We experimentally validate the relative preservation of T-cell expansion and activation following mTOR inhibition and significant suppression following MEK inhibition. Finally, we mechanistically demonstrated that *in vivo* tumor growth suppression following mTOR inhibition *in vivo* is CD8 cell dependent. These data have significant implications in the design of future experiments combing in these agents with immune-activating therapies.

## RESULTS

### MEK but not mTOR inhibition directly alters viability and function of MOC cells *in vitro*

**Figure 1:**
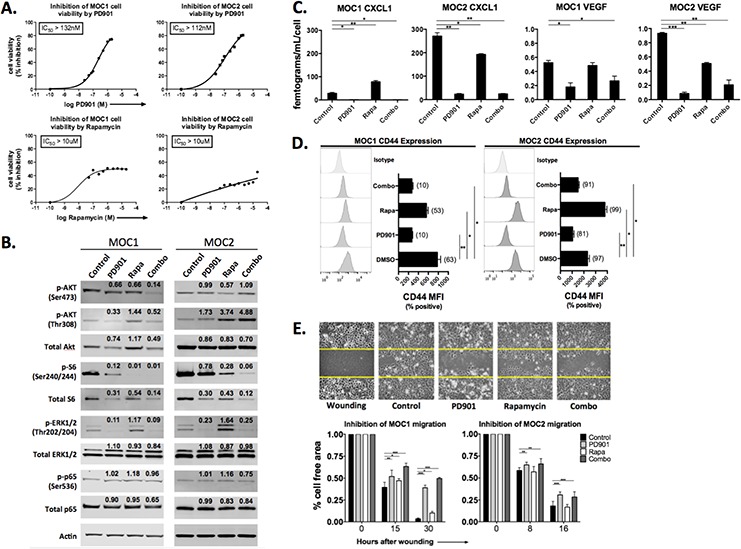
*In vitro* characterization of the effects of MEK and mTOR inhibition on MOC1 and 2 cells **A.** XTT assay data, following treatment with rapamycin or PD901 at the doses indicated for 48 hours. Results are representative of two independent experiments. **B.** western blot analysis of total and phospho-targets following treatment for 48 hours. Cells were treated with 1 μM rapamycin or 150 nM PD901 or both for 48 hours as indicated unless otherwise stated. Quantification of band density was normalized to actin. **C.** cytokine quantification via ELISA following treatment. Prior to ELISA, viable (trypan exclusion) cells were counted and cytokine levels were normalized to cell count to account for any drug-induced cytotoxicity. Combined results from two independent experiments are shown. **D.** flow cytometric analysis of CD44 expression following treatment. Nonviable cells were excluded from analysis via FSC/SCC gating and 7AAD staining (data not shown). Results representative of three independent experiments are shown. **E.** wounding assay following treatment. Representative MOC1 photomicrographs are 20x magnification, with yellow lines denoting baseline cell free region. Control consisted of equal volume of DMSO for all experiments. *p < 0.05, **p < 0.01, ***p < 0.001 for all experiments, analysis via one-way ANOVA with reference to control (DMSO treated) cells.

As Murine Oral Cancer (MOC) cells carry activating *Ras* mutations secondary to DMBA-induced carcinogenesis, we hypothesized that MOC cells would demonstrate variable sensitivity to rapamycin and PD901 treatment *in vitro*. Inhibition of MEK with PD901 resulted in efficient MOC cell killing with IC_50_s in the low nanomolar range for both MOC1 and MOC2 (132 nM and 112 nM, respectively, nonlinear regression analysis, Fig 1A). Conversely, mTOR inhibition with rapamycin failed to reach an IC_50_ dose in either cell line despite treatment with concentrations as high as 20 μM, indicating that MEK but not mTOR inhibition is directly cytotoxic to MOC1 and 2 cells.

We next validated on-target effects of both PD901 and rapamycin treatment *in vitro* via western blot analysis (Fig 1B). For these experiments, the IC_50_ dose of PD901, which falls within the serum concentration achieved in patients [15], was used. As no IC_50_ dose of rapamycin was achieved, a concentration of 1 μM was used for these experiments. Treatment with PD901 resulted in reduced phosphorylation of downstream ERK1/2 (T202/Y204) as expected, but also reduced phosphorylation of AKT (both residues) and S6K (S240/244) downstream of mTOR in MOC1 cells. Interestingly, MEK inhibition increased phosphorylation of AKT at the T308 residue and only modestly limited S6 phosphorylation in MOC2 cells, consistent with enhanced signaling though PI3K/PDK1 [16]. Rapamycin treatment suppressed phosphorylation of S6K as expected, but enhanced cross-activation of ERK to a greater degree in MOC2 than MOC1. Rapamycin also reduced phosphorylation of AKT at residue S473, consistent with mTORC2 suppression, but increased AKT T308 phosphorylation in MOC2 > MOC1, suggesting enhanced PI3K signaling through release of mTOR/S6K mediated negative-feedback inhibition [17]. Neither drug appeared to alter phosphorylation of RelA/p65 *in vitro*. Together, the compensatory activation of ERK and AKT provide a potential explanation for the limited anti-proliferative effects of rapamycin observed *in vitro*. Indeed, combination treatment induced significant suppression of both ERK and S6K phosphorylation. Thus, PD901 and rapamycin individually block phosphorylation of signaling proteins downstream of their expected targets, and observed compensatory activation of the untargeted pathway may be overcome with combination therapy.

HNSCC cells express angiogenic cytokines such as CXCL1 and VEGF downstream of PI3K and MAPK signaling [18, 19]. The production of both cytokines was significantly higher at baseline in MOC2 than MOC1 cells (*p* < 0.001, Fig 1C). In MOC1 cells, PD901 but not rapamycin consistently inhibited expression of CXCL1 and VEGF. In MOC2 cells, both rapamycin and PD901 significantly reduced CXCL1 and VEGF expression. In MOC cells, expression of adhesion and stem cell marker CD44 is tightly linked to ERK1/2 signaling [20]. Treatment with PD901 significantly reduced CD44 cell surface expression on both MOC1 and MOC2 cells, whereas rapamycin enhanced CD44 expression on MOC2 cells following treatment (Fig 1D). To assess alterations in migratory capacity, an *in vitro* wounding assay was performed (Fig 1E). PD901 but not rapamycin inhibition consistently reduced MOC cell migratory capacity in both MOC1 and 2 cells. Taken together, these data indicate that while both PD901 and rapamycin are able to block signaling through their respective targets, cytotoxicity is induced, cytokine and CD44 expression is altered and cellular migration is reduced following MEK but not mTOR inhibition *in vitro*. The predominant dependence of these cellular features on MEK signaling is consistent with the *Ras*-mutant genotype of MOC cells.

### Both MEK and mTOR inhibition significantly reduce MOC primary tumor growth *in vivo*

**Figure 2:**
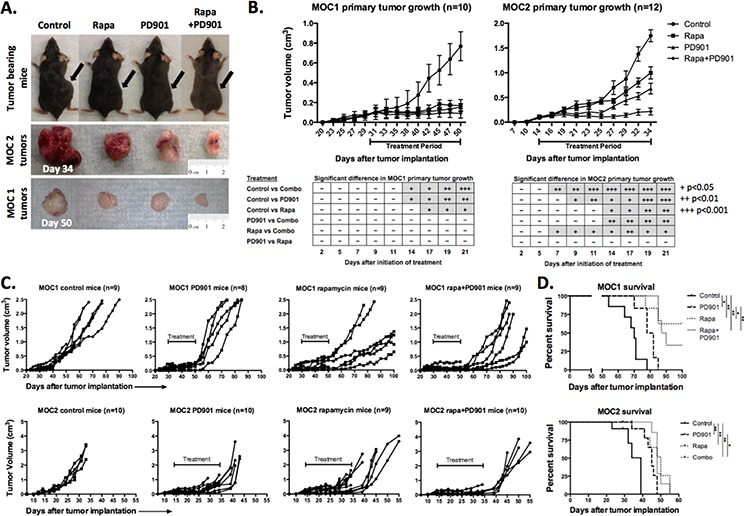
Alteration of primary tumor growth following MEK and mTOR inhibition *in vivo* **A.** Representative photographs of MOC1 (day 50) and MOC2 (day 34) tumor-bearing mice and corresponding excised tumors. **B.** Primary tumor growth curves for MOC1 and MOC2 tumors with indicated 21-day treatment period. MOC cells were transplanted and allowed to form tumors with a volume of 0.1 cm^3^ before initiation of treatment. Table reflects number of days after initiation of therapy needed to reach significant differences between growth curves, +*p* < 0.05, ++*p* < 0.01, +++*p* < 0.001 (parametric or non-parametric *t*-test for each time point). **C.** extended individual growth curves of tumor bearing mice plotting primary tumor growth after the completion of the treatment. Treatment period indicated on each plot. Extended growth data was derived from mice not euthanized for tissue at the end of the treatment period. All MOC2 tumor-bearing mice reached end-point criteria, whereas several rapamycin and combo treated MOC1 mice were alive at the end of the experiment (100 days) and were euthanized before end-point criteria were reached. **D.** survival data for MOC1 and MOC2 tumor-bearing mice (*p < 0.05, **p < 0.01, ***p < 0.001, Log-Rank/Mantel-Cox test).

Anti-cancer therapies often demonstrate effects in the complex tumor microenvironment beyond what is observed in culture. Above, we established that MEK significantly alters MOC1 and 2 cell viability and function. Conversely, mTOR inhibition appears to have little direct effect on MOC cells. To assess the effect of MEK and mTOR inhibition *in vivo*, MOC1 and MOC2 cells were transplanted into the flanks of immune-competent C57BL/6 mice and treated with PD901 and rapamycin alone or in combination. Fig 2A demonstrates MOC1 and MOC2 tumors excised following 21 days of treatment with drug or control (MOC1 day 50, MOC2 day 34), highlighting differences in tumor size and vascularity with treatment. In immunogenic MOC1 tumors, treatment with either drug alone significantly reduced primary tumor growth (Fig 2B, graph and table). Since either drug alone had significant effects, combining PD901 and rapamycin did not significantly enhance primary growth suppression in MOC1. Conversely, the combination of drugs significantly reduced primary tumor growth over either drug alone in poorly immunogenic MOC2 tumors. MEK blockade alone inhibited primary tumor growth more than mTOR blockade alone in MOC2 tumors, though the difference did not reach statistical significance. Combination rapamycin and PD901 was well tolerated with no treatment-associated weight loss while on treatment in either group, contrasted with MOC2 control mice that began to lose weight due to tumor associated cachexia toward the end of the treatment period (Supplementary Fig S1). These data suggest that while active MEK inhibition suppresses the growth of primary MOC1 and 2 tumors as expected, mTOR inhibition alone results in significant primary tumor growth inhibition in immunogenic MOC1 tumors despite there being no evidence of direct MOC1 cell cytotoxicity following mTOR inhibition *in vitro*.

### mTOR but not MEK inhibition induces a durable therapeutic tumor response in MOC tumors

Following completion of 21 days of treatment, mice not euthanized for tissue analysis were followed for post-treatment growth kinetics and survival. In MOC2 tumor-bearing mice, primary tumors grew rapidly following cessation of treatment with PD901 treated mice reaching euthanization criteria due to either primary tumor size (>2 cm diameter) or moribund status within one week (Figure 2C). MOC2 mice treated with rapamycin or combination demonstrated modest but statistically significant enhanced survival beyond control or PD901 treated mice (Figure 2D). Similarly, MOC1 tumor-bearing mice treated with PD901 demonstrated aggressive tumor growth rebound following cessation of treatment with statistically significant but minimal prolonged survival compared to control. Interestingly, the majority of MOC1 tumor-bearing mice treated with rapamycin alone or in combination with PD901 demonstrated durable primary growth suppression and survived to the end of the experiment (100 days). Combining PD901 with rapamycin appeared to attenuate the delayed growth and survival advantage over that observed with rapamycin alone. Again, despite having no direct cytotoxic effects *in vitro*, rapamycin appears to promote more durable primary tumor growth control and survival beyond that observed with PD901 in mice bearing RAS-mutant MOC tumors. Interestingly, this durable treatment effect of rapamycin is notably more pronounced in immunogenic MOC1 tumors than in poorly immunogenic MOC2 tumors.

### MEK but not mTOR inhibition consistently results in decreased proliferation and induction of apoptosis in MOC tumors

**Figure 3:**
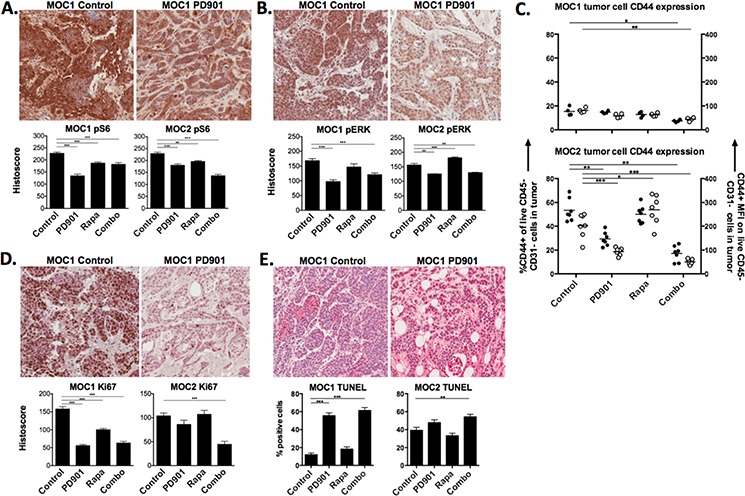
*In vivo* on-target effects of MEK and mTOR inhibition in MOC tumors Representative photomicrographs (20x) of immunohistochemical analysis of tumor sections from MOC1 and MOC2 tumors following 21 days of treatment with rapamycin and PD901 for **A.** S6K (S240/244), **B.** ERK1/2 (T202/Y204). **C.** flow cytometric analysis of cell surface CD44 expression on MOC1 and MOC2 tumors after treatment. Left y-axis represents % of tumor cells positive for CD44, right y-axis represents tumor cell CD44 mean fluorescence intensity (MFI). Significantly increased ERK1/2 phosphorylation and CD44 expression following rapamycin treatment alone in MOC2 tumors was reversed with combination treatment (B, C). **D.** analysis of proliferation of tumors cells following treatment via immunohistochemistry for Ki67. **E.** analysis of apoptosis of tumors cells following treatment via TUNEL assay. Shown are representative photomicrographs from MOC1 tumors. All stained section scoring was automated via Aperio ImageScope software analysis of ten 20x fields per section. Validation of the location of tumor cell nests and specific antibody staining was performed via pan-cytokeratin and isotype control staining, respectively, for each tumor and treatment condition (Supplementary Fig 2). *p < 0.05, **p < 0.01, ***p < 0.001 for all experiments, analysis via one-way ANOVA with reference to untreated (control) tumors.

To investigate mechanisms to explain the differences in response to therapy between MEK and mTOR inhibition in MOC tumor bearing mice, we wished to validate the on-target effects of PD901 and rapamycin treatment *in vivo*. Consistent with *in vitro* data, MEK inhibition with PD901 significantly reduced the phosphorylation of ERK and S6 in MOC1 and MOC2 tumors (Fig 3A, 3B). Rapamycin treatment suppressed phosphorylation of S6 and either did not alter (MOC1) or enhanced phosphorylation of ERK (MOC2), similar to effects *in vitro*. Enhanced phosphorylation of ERK following rapamycin treatment in MOC2 tumors was suppressed when combined with PD901. High baseline tumor cell CD44 expression in MOC2 was significantly reduced with PD901 but enhanced (by mean fluorescence intensity, MFI) with rapamycin treatment (Fig 3C). Again, this enhancement was reversed with combination therapy. Cumulatively, the alterations in phospho-targets and CD44 expression following treatment *in vivo* largely mirror those observed *in vitro* and suggest that both PD901 and rapamycin exert expected on-target effects on tumor cells *in vivo* and that compensatory activation of non-targeted pathways, which is pronounced in MOC2, can be reversed with combination therapy.

Functional consequences of MEK and mTOR inhibition were assessed by evaluating for tumor cell proliferation and apoptosis *in vivo*. Treatment with either drug alone significantly reduced proliferation measured via Ki67 staining in MOC1 tumors, whereas combination PD901 and rapamycin was needed to significantly decrease proliferation in MOC2 (Fig 3E). As measured by TUNEL assay, PD901 alone (MOC1) or in combination with rapamycin (MOC2) significantly induced apoptosis in tumor cells. These results suggest that despite both drugs demonstrating on-target signaling alterations, MEK but not mTOR inhibition consistently reduces tumor cell proliferation and induces tumor cell apoptosis in MOC tumors *in vivo*.

### MEK but not mTOR inhibition consistently alters tumor vascularity and expression of angiogenic cytokines in the tumor microenvironment

**Figure 4:**
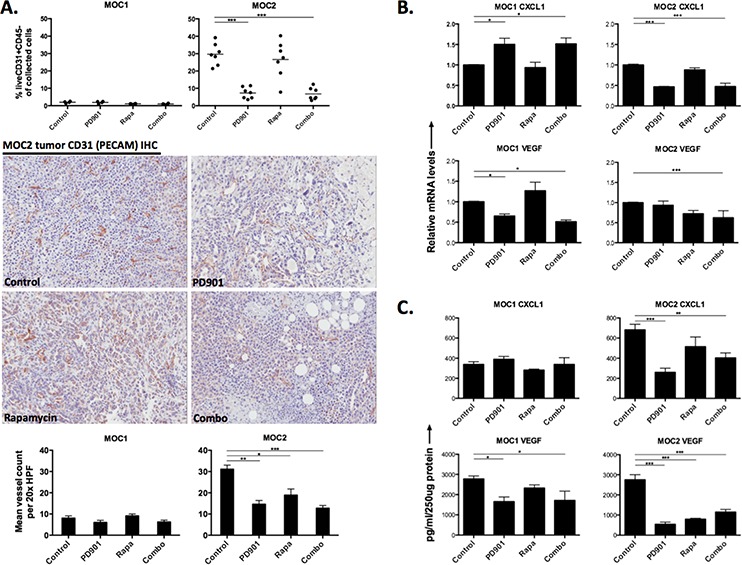
Variable alteration of *in vivo* tumor vascularity and angiogenic cytokine expression following MEK and mTOR inhibition **A.** flow cytometric analysis of CD45-CD31+ cells in MOC1 and MOC2 tumors along with representative 20x photomicrographs of MOC1 and MOC2 tumor CD31 IHC to visually assess CD31+ blood vessels. Mean vessel counts were calculated from eight 20x HPFs per tumor and treatment condition. Isotype control staining confirmed specific CD31 antibody staining. Expression of CD31 on MOC1 and MOC2 tumor cells was ruled out at the protein level *in vitro* with flow cytometry (data not shown). **B.** quantification of angiogenic cytokine RNA from MOC1 and MOC2 tumor lysates via RTPCR. **C.** quantification of angiogenic cytokine protein from MOC1 and MOC2 tumor lysates via ELISA. For RTPCR and ELISA, three separate tumors from each tumor and each treatment condition were assessed in technical triplicate. *p < 0.05, **p < 0.01, ***p < 0.001 for all experiments, analysis via one-way ANOVA with reference to untreated (control) tumors.

Consistent with their external appearance following resection (Fig 2A), baseline vascularity was higher in MOC2 compared to MOC1 (Fig 4A, CD31 flow cytometry and immunostaining). Treatment with PD901 but not rapamycin consistently and significantly reduced MOC2 tumor vascularity. Since a subset of CD31 cells in the tumors are also CD45+ and represent immature myeloid cells and not mature vascular cells, CD31 immunostaining was performed and revealed a pattern of intense mature vascularization in MOC2 tumors as measured by vessel count per HPF that was differentially altered by rapamycin and PD901 treatment. To determine if alterations in tumor vascularity correlated with changes in expression of angiogenic cytokines, RNA and protein levels of CXCL1 and VEGF were measured from MOC tumor lysates (Fig 4B, 4C). While baseline protein levels of VEGF were similar between MOC tumors, baseline CXCL1 was significantly higher in the MOC2 tumor microenvironment (*p* < 0.001). Treatment with PD901 reduced VEGF in both MOC1 and MOC2 tumors, but rapamycin led to reduced VEGF in MOC2 tumors only. Lower baseline CXCL1 expression in MOC1 tumors was largely unaffected by either drug. Conversely, higher baseline CXCL1 in MOC2 tumors was reduced modestly by rapamycin and significantly by PD901. Interestingly, while CXCL1 RNA and protein levels were congruent, significant differences between VEGF RNA and proteins levels suggests a greater degree of post-transcriptional regulation of VEGF in MOC tumors. Further, cytokine measurements from MOC tumor lysates and from MOC cells *in vitro* differ significantly and suggest stromal or other microenvironment contributions to cytokine levels *in vivo*. Again, MEK but not mTOR inhibition results in consistently altered tumor vascularity and angiogenic cytokine/chemokine expression in MOC tumors. Cumulatively, the above experiments demonstrating altered tumor cell viability, proliferation, CD44 expression and MOC tumor vascularity and chemokine expression following MEK inhibition do not explain the durable treatment response observed in MOC1 but not MOC2 tumors following withdrawal of mTOR inhibition.

### Differences in baseline and effects of MEK and mTOR inhibition on the infiltration and activation of innate immune cell subsets in immunogenic and non-immunogenic MOC tumors

**Figure 5:**
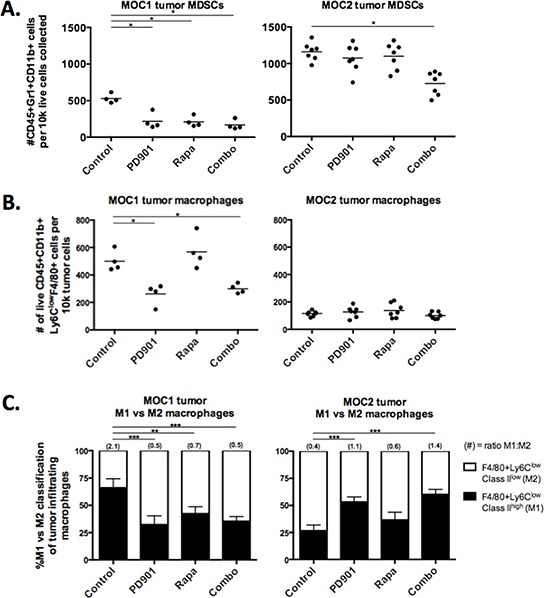
MEK and mTOR inhibition variably alter myeloid cell tumor infiltration and phenotype Flow cytometric analysis of tumor infiltrating myeloid cells in MOC1 and MOC2 tumors. Dead cells excluded via 7AAD staining in all experiments. Each data point represents a separate tumor and reported values are per 1 × 10^5^ total collected cells. **A.** infiltration of CD45+Gr1+CD11b+ MDSCs in rapamycin and PD901 treated tumors. **B.** infiltration of CD45+CD11b+Ly6C^low^F4/80+ macrophages in rapamycin and PD901 treated tumors. **C.** CD45+CD11b+Ly6C^low^F4/80+ macrophages were phenotyped by cell surface MHC class II expression (and CD206 surface expression, see Suppl Fig 4B). We classified M1s as MHC Class II high and M2s as MHC Class II low. Changes in the M1/M2 ratio following treatment with rapamycin and PD901 are shown. Tissues used for analysis are from one *in vivo* experiment in MOC1 and two independent experiments in MOC2. *p < 0.05, **p < 0.01, ***p < 0.001 for all experiments, analysis via one-way ANOVA with reference to untreated (control) tumors.

Given that mTOR inhibition results in durable primary tumor growth inhibition and prolonged survival of immunogenic MOC1 tumor bearing mice, yet does not appear to greatly alter MOC1 tumor cells directly, we hypothesized MEK and mTOR inhibition may differentially modulate the MOC tumor microenvironment. Following MOC1 and 2 transplantation into C57BL/6 mice, we assessed baseline differences and alterations in tumor immune infiltration following treatment via flow cytometry. Whereas Gr1+CD11b+ immature myeloid derived suppressor cells (MDSCs) can mediate T-cell immunosuppression [21], subsets of mature F4/80+ tumor-associated macrophages (TAMs) can mediate anti-tumor immunity [22]. Systemically, MOC tumor-bearing mice demonstrated significantly higher accumulation of splenic MDSCs compared to non-tumor bearing mice, and this splenic MDSC accumulation did not change with PD901 or rapamycin treatment (Supplementary Fig S3). MOC2 tumors demonstrated significantly higher baseline MDSC recruitment than MOC1 tumors (*p* < 0.001, Fig 5A, gating strategy Supplementary Fig S4A). High baseline MDSC accumulation in MOC2 was reduced significantly following combination therapy but not with either PD901 or rapamycin alone. In MOC1 tumors, low baseline MDSC accumulation was reduced with rapamycin or PD901 alone or in combination.

In contrast to MDSCs, mature tumor-infiltrating macrophages were significantly higher in MOC1 than MOC2 (*p* < 0.001, Fig 5B, gating strategy Supplementary Fig S4B). Treatment with PD901 but not rapamycin significantly reduced accumulation of TAMs in MOC1 tumors, whereas low baseline TAM accumulation in MOC2 tumors did not change. We further characterized TAMs as tumor-suppressing M1 or tumor-promoting M2 phenotypes based upon cell surface MHC class II and CD206 expression (Supplementary Fig S4B). Evaluated as a ratio of M1:M2, MOC1 TAMs are a predominantly M1 and MOC2 TAMs are a predominantly M2 phenotype (Fig 5C). PD901 reversed TAM phenotype in MOC1 tumors with a shift from M1 to M2 phenotype and reduced recruitment of M2 TAMs in MOC2 tumors, leading to a M1:M2 ratio reversal from baseline in both tumors. Rapamycin treatment altered the M1:M2 ratio in MOC1 tumors as well but did not significantly alter the MOC2 tumor M1:M2 ratio. Thus, while PD901 reduced macrophage infiltration and skewed macrophages toward the M2 phenotype in MOC1 tumors, it increased the M1:M2 TAM ratio and reduced MDSC accumulation when combined with rapamycin in MOC2 tumors. Together, these data are interesting, but do not convincingly provide a mechanism for mTOR inhibition induced durable MOC1 tumor control.

### Differences in baseline and effects of PD901 and rapamycin on the infiltration and activation of T-cell subsets in immunogenic and non-immunogenic MOC tumors

**Figure 6:**
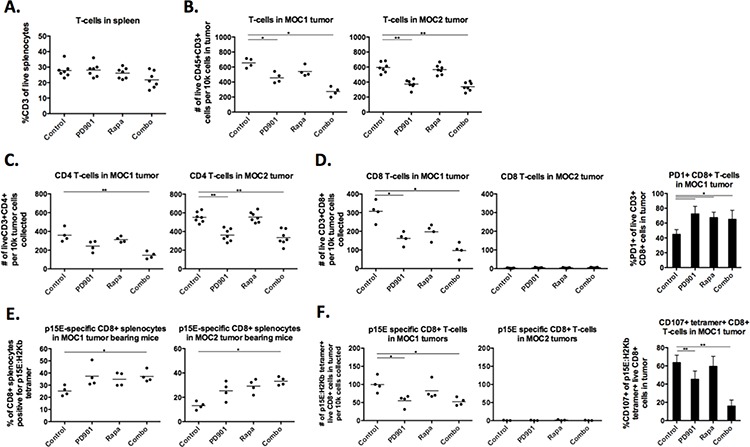
MEK and mTOR inhibition variably alter systemic and tumor-infiltrating lymphocytes Flow cytometric analysis of tumor infiltrating myeloid cells in MOC1 and MOC2 tumors. Dead cells excluded via 7AAD staining in all experiments. Each data point represents a separate tumor and reported values are per 1 × 10^5^ total collected cells. **A.** CD3 cells in the spleens of MOC1 and MOC2 tumor-bearing mice treated with rapamycin and PD901. **B.** infiltration of CD45+CD3+ cells into treated MOC1 and MOC2 tumors. **C.** infiltration of CD45+CD3+CD4+ cells into treated MOC1 and MOC2 tumors. **D.** infiltration of CD45+CD3+CD8+ cells into treated MOC1 and MOC2 tumors, along with MOC1 tumor-infiltrating CD8 cell surface PD1 expression. **E.** % positivity of CD8+ splenocytes specific for an H2K^b^-restricted p15E antigen (KSPWFTTL) in MOC1 and MOC2 tumor-bearing mice via tetramer staining. **F.** infiltration of CD45+CD3+CD8+tetramer+ cells into treated MOC1 and MOC2 tumors, along with CD107a staining on these tumor-infiltrating tetramer+CD8+ cells as a measure of activation and degranulation. Tissues used for analysis are from one *in vivo* experiment in MOC1 and two experiments in MOC2. *p < 0.05, **p < 0.01, ***p < 0.001 for all experiments, analysis via one-way ANOVA with reference to untreated (control) tumors.

We next hypothesized that MEK and mTOR inhibition may exert different effects on the development of adaptive immunity within the MOC tumor microenvironment. We systematically evaluated the infiltration and activation status of specific T-cell subsets (gating strategy Supplementary Fig 5A) following MEK and mTOR inhibition. Systemic (splenic) T-cells levels were not significantly altered with any treatment (Fig 6A). However, PD901 consistently induced a reduction in the number of infiltrating CD3+ T-cells in MOC1 and MOC2 tumors (Fig 6B). At baseline, an even mix of CD4 and CD8 T-cells infiltrate into MOC1 tumors (Fig 6C, 6D). While similar numbers of CD3+ T-cells infiltrate MOC2 tumors, these are nearly exclusively CD4 with very few infiltrating CD8 cells. In both tumors, CD4 cells were reduced following PD901 treatment in a similar pattern to CD3 cells. For CD8 cells, PD901 significantly reduced CD8 cell infiltration into MOC1 tumors (Fig 6D). Rapamycin trended toward the same effect but did not reach statistical significance. Neither drug enhanced CD8 cell recruitment into MOC2 tumors. As a measure of CD8 cell activation, we measured cell surface PD-1 expression and found that rapamycin and PD901 alone or in combination significantly induced PD-1 expression on MOC1 infiltrating CD8 cells. This enhanced PD1 expression was not seen on splenic CD8 cells following treatment (Supplementary Fig 5B), suggesting an enhanced degree of CD8 antigen exposure within the MOC1 tumor microenvironment [23].

To evaluate the functional effects on tumor specific immunity, we examined specific CD8+ responses to the tumor-associated antigen and endogenous retroviral envelope protein p15E that is known to harbor an H-2K^b^-restricted antigenic epitope (KSPWFTTL) and is expressed in MOC cells. To evaluate changes in p15E antigen specific CD8 T-cells, an H-2K^b^:KSPWFTTL tetramer was used to stain for p15E specific CD8 T-cells [24, 25]. Baseline tetramer positivity of splenic CD8 cells was higher in MOC1 than MOC2 (Fig 6E), indicating more CD8 T-cell exposure to p15E antigen in MOC1 tumor bearing mice. Treatment with rapamycin or PD901 alone tended to increase the number of p15E-specific T-cells without affecting the overall percentage of CD8 cells in the spleen (Supplementary Fig 5C), and this reached significance with combination therapy in mice bearing both MOC1 and 2 tumors indicating that targeted therapy was enhancing exposure to antigen and expansion of antigen-specific CD8 T-cells. Despite this systemic effect, PD901 but not rapamycin reduced tumor infiltration of p15E-specific CD8 cells in MOC1 tumors and neither drug induced accumulation of p15E-specific CD8 cells into MOC2 tumors (Fig 6F). Using CD107a expression as a specific marker of activation/degranulation in antigen specific CD8 cells in MOC1 tumors [26], staining suggested a high degree of tetramer+ CD8 cell activation at baseline within tumors that was not observed in tetramer+ splenocytes from MOC1 tumor-bearing mice (Supplementary Fig 5D). MEK but not mTOR inhibition significantly reduced CD107a staining of infiltrating p15E-specific CD8 cells in MOC1 tumors indicating inhibition of antigen-specific CD8 T-cell degranulation following MEK inhibition.

For completeness, we examined for the presence of CD4+FoxP3+ Treg cells in both models (Supplementary Fig S6). Consistent with differences in their immunogenic phenotypes, MOC1 tumors exhibited a lower baseline level of Tregs compared to MOC2 tumors. Rapamycin modestly increased the number of infiltrating FoxP3+ CD4 cells. Alternatively, PD901 decreased FoxP3+ CD4 cells in MOC2 tumors. Interestingly, 20–30% of FoxP3+ CD4 cells in both tumors were CD25+ (compared with 70–80% of all splenic CD4+FoxP3+ Tregs), indicating that the majority of Tregs in MOC tumors are induced and not of thymic origin [27]. Treg CD25 cell surface expression was substantially reduced following rapamycin or PD901 treatment.

Cumulatively, these data suggest that MEK inhibition significantly inhibits the presence and activation status of T-cells in MOC tumors. In the case of MOC1, these effects tend to be detrimental with reduced infiltration of both total and antigen-specific CD8 cells as well as reduced antigen-specific degranulation of antigen-specific CD8 cells as measured by CD107a cell surface expression. Thus, mTOR inhibition appears to preserve while MEK inhibition appears to inhibit antigen-specific T-cell responses in immunogenic MOC1 tumors, potentially explaining the durable responses observed following mTOR but not MEK inhibition in immunogenic MOC1 tumor bearing mice.

### MEK inhibition significantly suppresses CD3/28 activated CD4 and CD8 T-cell expansion and function

**Figure 7:**
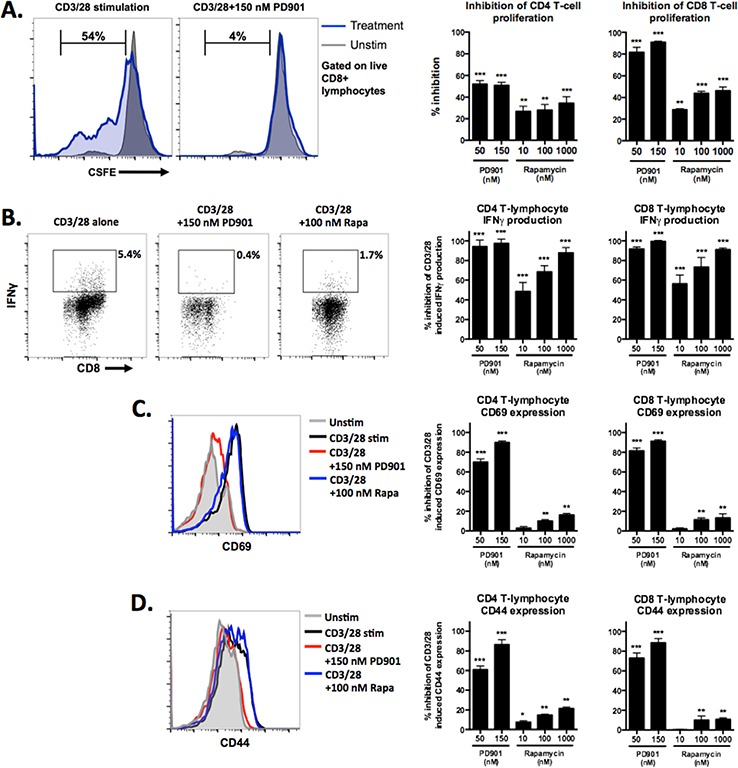
Effects of MEK and mTOR inhibition on the proliferation and activation of stimulated T-cells Flow cytometric analysis of sorted T-cells. **A.** CFSE labeled T-cells were stimulated with CD3/28 microbeads for 7 days in the presence of control or drug at the doses indicated. Representative histograms illustrating unstimulated (grey) and stimulated (blue) patterns of CFSE intensity on the left, with quantification of % inhibition relative to the maximum percentage of CFSE+ cells that move out of the parent population with CD3/28 stimulation alone using the formula [(expt – spon)/(max – spon)] on the right. **B.** representative dot plots of CD8 T-cells expressing IFNγ in response to CD3/28 stimulation with and without drug on the left, calculated % inhibition of the maximum percentage of IFNγ expressing cells with CD3/28 stimulation alone on the right. **C.** and **D.** representative histograms demonstrating CD69 and CD44 expression, respectively, on the surface of T-cells in response to CD3/28 stimulation with and without drug on the left, calculated % inhibition of the maximum MFI for each marker with CD3/28 stimulation alone on the right. Experiments were replicated three times. *p < 0.05, **p < 0.01, ***p < 0.001 for all experiments, analysis via one-way ANOVA with reference to the inhibition of the % maximum for each experiment.

Based on the above *in vivo* data suggesting that MEK inhibition potentially reduces the number and activation status of T-cells within the MOC tumor microenvironment, we experimentally measured the effect of MEK and mTOR inhibition on T-cells *in vitro*. At *in vitro* doses of drug well within those achieved in serum with treatment in mice and humans, MEK inhibition more significantly reduces CD4 and CD8 T-cell proliferation following physiologic stimulation with CD3/28 microbeads (Fig 7A). Similarly, IFNγ production and expression of activation and antigen-exposure markers CD69 and CD44 were significantly suppressed following T-cell activation in the presence of MEK but not mTOR inhibition (Fig 7B–7D). These data support our *in vivo* findings in immunogenic MOC1 tumors and indicate that, whereas MEK inhibition significantly suppresses T-cell expansion and function, mTOR inhibition relatively preserves it.

### Primary tumor growth inhibition following mTOR inhibition in immunogenic MOC1 tumors is CD8+ cell dependent

**Figure 8:**
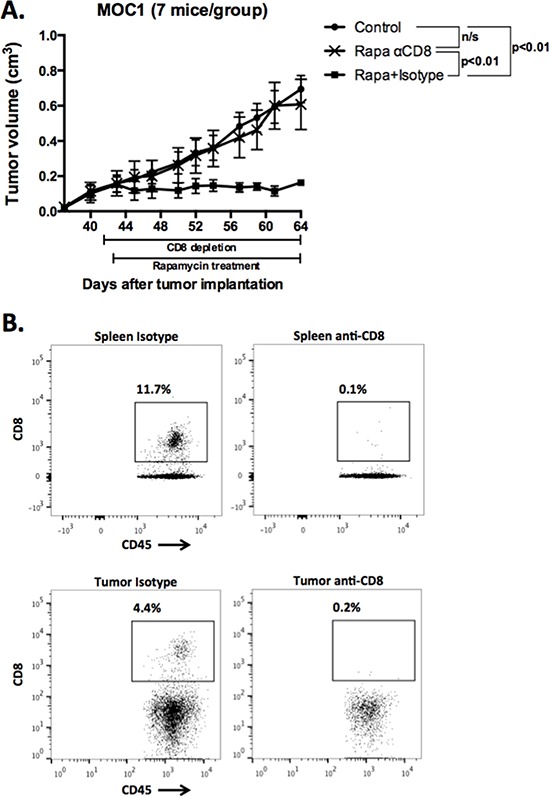
Abrogation of MOC1 tumor growth inhibition following mTOR inhibition with systemic CD8 cell depletion **A.** MOC1 cells were transplanted and allowed to form tumors with a volume of 0.1 cm3. Depletion of CD8 cells with 200 μg anti-CD8 antibody IP twice weekly was started one day before initiation of mTOR inhibition with rapamycin. Injection of a non-specific isotype antibody served as control. **B.** At the completion of the experiment, spleen and tumor tissues were harvest and flow cytometry performed to validate the depletion of CD8 cells both peripherally and in the tumor. Representative dot plots of spleen (top panels) and tumor tissue (bottom panels) are shown.

Given the lack of direct cytotoxic effects of mTOR inhibition on MOC1 cells, yet the durable tumor control following cessation of treatment and measured preservation of antigen-specific T-cell responses in MOC1 treated tumors, we hypothesized that the primary mechanism of rapamycin-induced growth delay was related to tumor immunity. To test this, we treated MOC1 tumor bearing mice with the same rapamycin protocol with and without antibody-based systemic CD8 cell depletion. Fig 8A demonstrates that the primary growth inhibition of MOC1 tumors with mTOR inhibition is nearly completely abrogated with CD8 cell depletion compared to isotype control. Validation of complete systemic and intra-tumoral CD8 T-cell depletion is shown in Fig 8B. This data strongly suggests that the primary tumor growth inhibition and subsequent durable tumor control and improved survival in MOC1 tumor bearing mice following rapamycin treatment is due to preserved CD8 T-cell responses, whereas the lack of such responses following PD901 treatment is due to MEK-induced inhibition of adaptive immunity.

## DISCUSSION

Here, we utilize newly described syngeneic MOC models of murine oral cavity cancer [20] to demonstrate differential effects of the mTOR inhibitor rapamycin and the MEK1/2 inhibitor PD901 alone or in combination on oncogenic signaling, tumor responses and survival and immune modulation in highly immunogenic (MOC1) and poorly immunogenic (MOC2) tumors. MEK but not mTOR inhibition *in vitro* induced cytotoxicity, reduced inflammatory cytokine secretion and CD44 expression and reduced migratory capacity in MOC1 and MOC2 cells, consistent with the *Ras*-driver mutations shared by these carcinomas. Remarkably, different effects of these drugs were observed in MOC derived tumors *in vivo*. Both drugs alone or in combination significantly inhibited primary tumor growth, indicating a role for targeting MAPK and/or PI3K/mTOR signaling *in vivo*. Consistent with direct anti-proliferative and cytotoxic effects observed *in vitro*, MEK inhibition suppressed growth during the 21 days of treatment, but withdrawal led to rapid rebound of primary tumor growth and minimal improvement in survival in both MOC1 and MOC2 tumor-bearing mice. While mTOR inhibition had limited effects *in vitro*, rapamycin potently inhibited tumor growth *in vivo* and subsequent drug withdrawal after 21 days of treatment was followed by a more durable response in a significant subset of tumor bearing mice with resulting prolonged survival compared to MEK inhibition. This treatment durability after withdrawal of therapy and survival effect of mTOR inhibition relative to MEK inhibition appeared to be more pronounced in immunogenic MOC1 tumor bearing mice compared to MOC2 tumor bearing mice.

Several observations from this and prior studies support the hypothesis that the enhanced durability of primary tumor control and prolonged survival in rapamycin treated MOC1 tumors could be due to differences in basal and drug effects on tumor microenvironment immune infiltration and/or activation. This differential effect did not appear to be due to enhanced *in vivo* inhibition of phospho-targets, reduced cell surface CD44 expression (known to correlate with MOC cell aggressiveness [20]), reduced tumor cell proliferation, reduced tumor vascularization or expression of angiogenic cytokines or enhanced tumor cell apoptosis, all of which were similarly or more significantly altered by MEK inhibition. The use of a syngeneic model allowed assessment of the infiltration and functional status of cells of innate and adaptive immunity that is limited in immunodeficient models. Treatment with PD901 alone or in combination was not associated with durable survival in immunogenic MOC1 tumors. PD901 inhibited tumor infiltration of mature macrophages and reversed TAM polarization toward an M2 phenotype in MOC1 tumors. Mature macrophages producing TNF may be important in the inhibition of immunogenic tumors such as MOC1 [28]. Importantly, PD901 treatment appeared to inhibit total CD4 and CD8 T-cell and antigen-specific CD8 T-cell infiltration into MOC1 tumors as well as antigen-specific T-cell activation/degranulation as assessed by CD107a cell surface staining. In response to these results, we experimentally measured the relative suppression of activated T-cell expansion and activation, as measured by IFNγ secretion and CD69/CD44 expression, and found potent suppression following MEK inhibition. Thus, blockade of adaptive T-cell responses and functional immunity following PD901 treatment provide a mechanistic explanation for the rapid rebound of primary tumor growth in MOC1 tumor bearing mice following cessation of treatment. To validate that the primary mechanism of rapamycin-induced MOC1 primary tumor growth inhibition was immunogenic, given the lack of measurable alteration in MOC1 cancer cell function, we depleted CD8 Cells in MOC1 tumor bearing mice and found that this essentially completely abrogated the growth inhibitory effects of rapamycin. To our knowledge, this study is the first to demonstrate a primarily immune-dependent mechanism of rapamycin-induced tumor growth inhibition. In MOC2 tumors, which have very high immunosuppressive myeloid and lymphoid immune infiltration at baseline, several desirable changes were observed including reduced MDSC accumulation with combination therapy and a skewing of TAMs toward an M1 phenotype and reduced Treg tumor infiltration following MEK inhibition. However, we were unable to induce an adaptive response (recruitment of CD8 T-cells) within the tumor with either drug despite apparent enhancement of systemic antigen-specific CD8 T-cells. Investigations into how to best enhance the immunogenicity of MOC2 tumors are underway.

Signaling through the MAPK pathway is known to be critical to T-cell function [9, 29], and our findings support the work of others demonstrating blockade of T-cell proliferation, cytokine expression and antigen-specific expansion *in vitro* and *in vivo* following MEK but not Raf inhibition [30, 31] that may be model dependent. Our data strongly supports the need for further investigation into the effects of MEK inhibition in immunogenic tumors, where blockade of T-cell function may abrogate an existing anti-tumor immune response. Similarly, despite that rapamycin is used for immunosuppression following solid organ transplantation, rapamycin has shown anti-tumor activity in a variety of solid tumor types [32]. Rapamycin enhances development of memory T-cell populations, perhaps at the expense of effector populations, and induces development of FoxP3+ Tregs [33–35]. These findings with ours support the context dependent immunosuppressive properties of rapamycin. Together with evidence that combination mTOR and MEK blockade enhanced on-treatment growth suppression of non-immunogenic MOC2 tumors, our findings of altered tumor immunity following MEK inhibition and preserved immunity following mTOR inhibition in immunogenic MOC1 tumors suggests the need for individual patient anti-cancer therapies to be tailored not only to identified oncogenic pathways but also to the immune status of the tumor. Patients with immunogenic tumors may benefit more from therapies that preserve existing anti-tumor immune responses, especially when combined with an immunotherapy such as a tumor vaccines or checkpoint inhibitor. Approximately 50–70% of patients with HNSCCs harbor immunogenic tumors as defined by CD8 T-cell tumor infiltration [13], a lymphocyte “active” signature [3], and PD-L1 expression [12] and would be modeled by MOC1 tumors. Conversely, poorly immunogenic tumors may be modeled by MOC2 tumors, for which the relative paucity of antigenic genomic alterations and high degree of local immune suppression may limit the effectiveness of immune-stimulating therapies.

For poorly-immunogenic tumors such as MOC2, with genomic alterations co-activating MAPK and PI3K/mTOR pathways, combination MEK and mTOR inhibition maybe needed. Compensatory MAPK pathway activation and CD44 cell surface expression following mTOR inhibition alone was observed both *in vitro* and *in vivo* in MOC2 cells and tumors. This was reversed with combination therapy and may explain in part why combination treatment was needed to effectively suppress on-treatment primary tumor growth in MOC2 tumors. The concept of co-activated signaling pathway enhancement as a mechanism of targeted therapy resistance is well documented, and our data validates the principle of using combination therapy to block compensatory signaling through non-targeted but co-activated oncogenic pathways [5, 36]. The combination of rapamycin and PD901 was well tolerated in this model with no appreciable weight loss or significant side effects from treatment.

Several limitations to our study exist. First, cell lines in the MOC model are RAS mutant and PIK3CA wild-type, similar to only a small subset of human HNSCC [3]. However, growth factor receptors and Ras activate PI3K, and this model exhibits co-activation of both PI3K/mTOR and MAPK pathways similar to the majority of human HNSCC. Indeed, the overall genomic landscape between MOC tumors and human HNSCC is highly conserved [37]. Additionally, we observed durable, immune-based responses following mTOR inhibition in MOC tumors *in vivo* despite the fact that they are *Ras* mutant. Next, tumor tissue used for the immune analyses were studied after prolonged treatment, and it is possible that the immune profile of MOC tumors is different in earlier stages of tumor progression and treatment. Additionally, mTOR and MEK inhibition may alter the interaction between tumor and other immune or stromal cells, such as cancer-associated fibroblasts, that were not evaluated here. These models may enable further experiments exploring different mechanisms of immune activation or suppression following treatment with different therapies, which are ongoing in our laboratory.

In summary, this study demonstrates the utility of the mTOR inhibitor rapamycin and the MEK1/2 inhibitor PD901 in syngeneic models of oral cavity cancer. Not only do we validate the on-target effects of each drug both *in vitro* and *in vivo*, we demonstrate different mechanisms of primary tumor growth control between immunogenic MOC1 and poorly immunogenic MOC2 tumors. We experimentally show that MEK inhibition inhibits but mTOR inhibition relatively preserves anti-tumor T-cell responses both via tetramer-based flow cytometry in MOC1 tumor tissue and in an *in vitro* T-cell proliferation and activation assay. Our finding that mTOR inhibition leads to a CD8 cell dependent anti-tumor response in RAS-mutant, immunogenic MOC1 tumors is novel and suggests that mTOR targeting therapies may be useful in multiple tumor types regardless of underlying driver mutations. Further, these results have significant implications for future clinical trial planning involving combinations of these targeted therapies, as well as highlight the need for enhanced understanding of how different anti-cancer therapies alter tumor immunity to allow the rational design of anti-cancer and immunotherapy combinations.

## MATERIALS AND METHODS

### Cell lines

Murine Oral Cancer (MOC) cells were generated and maintained in culture in complete media as described previously [11, 20]. Treatment of MOC cells with pharmaceutical grade rapamycin (LC Labs, Woburn MA) and PD0325901 (PD901, SelleckChem, Houston, TX) was performed as indicated.

### XTT assays

MOC1 and MOC2 cells were seeded at 10^3^ cells/well (96-well plate) in DMEM with 10% heat-inactivated fetal calf serum and 1% penicillin-streptomycin. After 24 hours, cells were treated with varying concentrations of drug or vehicle and assessed for alteration in viability utilizing the XTT assay (Roche Diagnostics, Indianapolis, IN).

### Western blots

MOC cell lines were grown in 10 cm culture plates and treated with control or drug at the indicated concentrations for 48 hours. Following lysis, total protein levels were measured and blotting was performed using standard technique with actin control. All antibodies were purchased from Cell Signaling Technology (Danvers, MA).

### ELISA

#### In vitro

MOC1 and MOC2 cells were seeded at 10^5^ cells/well (6-well plate) in complete media. After 24 hours, cells were treated with varying concentrations of drug or vehicle as indicated. ELISA kits were purchased from R&D Systems (Minneapolis, MN) and used per manufacturer's protocol. Reported cytokine expression levels were normalized for viable cell count.

#### In vivo

Approximately 20 mg of snap frozen tissue was lysed in the presence of protease and phosphatase inhibitors on the Qiagen TissueLyser II (30 Hz × 2 min) with stainless steel disruption beads. Following determination of total protein levels, samples were diluted to standardized concentrations and ELISA kits were used as above.

### Wounding assay

MOC1 and MOC2 cells were seeded at 10^5^ cells/well (6-well plate) in complete media, allowed to reach 90% confluence, and pipette tips were used to generate perpendicular linear cell free wounds. Time 0 pictures were taken immediately following the addition of treated media. For each treatment condition and time point, a total of 8 images were captured and cell-free area was quantified using ImageStudioLite software.

### Mice and *in vivo* experiments

Experiments were carried out using 8–10 week old female C57BL/6 mice purchased from the National Cancer Institute (NCI) Animal Production Facility in Frederick, MD. All animal studies were approved by the National Institute of Deafness and Other Communication Disorders Animal Care and Use Committee (ASP1364–14). To generate tumors, 1 × 10^6^ MOC1 or 1 × 10^5^ MOC2 cells were transplanted subcutaneously in the right flank. Tumors were allowed to engraft and reach a size of 0.1 cm^3^ before treatment. Mice treated with rapamycin were administered a loading dose of 4.5 mg/kg via intraperitoneal (IP) injection, followed by 1.5 mg/kg via IP injection every other day for 21 days. Mice treated with PD901 were administered 1.5 mg/kg via oral gavage (OG) daily for 21 days. Control mice were treated with drug carriers alone (2% ethanol, 5.2% tween 80 and 5.2% PEG 400 in water for rapamycin and 0.5% HPMC and 0.2% tween 80 in water for PD901). Following 21 days of therapy, treated and control tumor-bearing mice were euthanized and tumors were surgically removed. Tumor tissue used for RT-PCR or ELISA was snap frozen, tissue used for immunohistochemistry was fixed with 4% formalin for 24 hours and stored in 70% ethanol until use, and tissue for flow cytometric analysis was used fresh. In some experiments, systemic CD8 cell depletion was performed via IP injection of 200 μg of anti-CD8 antibody (clone YTS169.4, BioXCell, Lebanon, NH) twice weekly.

### Immunohistochemistry

Formalin-fixed, paraffin-embedded tumors were sectioned (6 micron thickness) by a third party vendor (Histoserv, Germantown, MD). Sections were deparaffinized with Histoclear and rehydrated with an ethanol gradient. Following quench of endogenous peroxidase with diluted hydrogen peroxide, and blocking with serum of the species of the secondary antibody, primary antibodies at pre-titrated concentrations were incubated overnight at 4C. All primary antibodies with the exception of CD31 (Abcam, Cambridge, MA) and the isotype controls (Biolegend, San Diego, CA) were from Cell Signaling Technology. Following incubation for 1 hour with a biotinylated secondary antibody, signal was amplified with an ABC kit per manufacturer's protocol and colorimetric staining was developed with DAB (Vector Labs, Burlingame, CA). Washes between each step were performed with a PBS based wash solution. Developed samples were counterstained with Hematoxylin QS (Vector Labs, cat# H-3404) and coverslips were mounted with Permount. Whole slides were digitized for analysis using the Aperio ScanScope CS system and quantified using the Aperio ImageScope Viewer software's positive pixel count v9 algorithm or nuclear staining algorithm. Pancytokeratin staining was performed on each tissue block to ensure differentiation of tumor and stromal tissue, and isotype control stains were performed with each assay to ensure specific antibody staining (Suppl Fig 2).

### TUNEL

Formalin-fixed, paraffin-embedded tumor sections were stained via a standard TUNEL assay protocol using an alkaline phosphatase enzyme label and a fuchsin (red) substrate.

### RTPCR

Tumor lysates were generated using the Tissue Lyser II and total RNA was purified using the RNEasy Mini Kit (Qiagen, Valencia, CA) per the manufacturer's protocol. cDNA was synthesized utilizing a High Capacity cDNA Reverse Transcription Kit (Life Technologies, Waltham, MA). Taqman Single Tube primers and a Universal PCR Master Mix (Life Technologies) were used to assess the relative expression of target genes in comparison to GAPDH on a Viia7 qPCR analyzer (Applied Biosystems, Carlsbad, CA).

### Flow cytometry

#### In vitro

MOC cells were treated as indicated and passed through a 40 μM filter to generate a single cell suspension. Nonspecific binding was blocked using an anti-mouse CD16/32 antibody (Biolegend, San Diego, CA) and cells were stained with a flourophore-conjugated CD44 clone IM7 antibody. Washes between steps was performed with a 1% BSA in 1xPBS solution. Dead cells were excluded via 7AAD staining in all experiments.

#### In vivo

Following *in vivo* treatment as indicated, fresh tumor tissue was minced into 1 mm pieces and digested into a single suspension using the murine tumor dissociation kit from Miltenyi Biotech (Auburn, CA) per the manufacturer's protocol. Both chemical and mechanical (gentleMACS, Miltenyi) dissociation techniques were utilized with consistent cell viability of 70% or greater. After filtering the tumor suspension through a 100 μM filter and several washes with a 1% BSA in 1xPBS solution, cell surface staining was performed using flourophore conjugated anti-mouse CD45.2 clone 104, Gr1 clone RB6–8C5, Cd11b clone M1/70, Ly6C clone HK1.4, F4/80 clone BM8, I-A/I-E clone M5/114.15.2, CD206 clone C068C2, CD3 clone 145–2C11, CD4 clone GK1.5, CD8 clone 53–6.7 or KT15, CD25 clone PC61, PD1 CLONE RMP1–30, CD107a clone 1D4B antibodies (Biolegend) as indicated. H2Kb:KSPWFTTL tetramer was purchased from MBL (Woburn, MA). Use of anti-CD8 antibody close KT15 was required with tetramer staining sue to epitope compatibility, and specificity of tetramer staining was validates with KSPWFTTL peptide vaccination of naïve C57BL/6 mice (data not shown). Intracellular staining for FoxP3 in CD4+ T-lymphocytes was performed using the eBioscience (San Diego, CA) Treg staining kit #1 per manufacturer's protocol. Specific staining of each antibody used was validated with the use of isotype control antibodies and a “fluorescence minus one” method of antibody combination validation. Dead cells were excluded with 7AAD staining if cell surface staining only and via LIVE/DEAD fixable viability dye staining (Life Technologies) for intracellular staining. Data was acquired on a FACSCanto using FACSDiva software (BD Biosciences, San Diego, CA) and analyzed on FlowJo software vX.0.7r2 (Ashland, OR).

### *In vitro* T-cell assays

Murine splenocytes were sorted from whole spleens via magnetic separation using an “untouched” T-cell purification approach (autoMACS, Miltenyi). T-cells were incubated with CD3/CD28 coated microbeads (Mouse T-activator Dynabeads, Life Technologies) at a 1:1 cell to bead ratio in complete T-cell media. For T-cell proliferation experiments, T-cells were labeled with 5 μM CSFE and incubated with CD3/28 microbeads for 7 days with either drug or control. Flow cytometry was then used to measure loss of CFSE signal in proliferating T-cells. For T-cell activation assays, sorted T-cells were incubated with CD3/28 microbeads for 24 hours, then flow cytometry-based assays were performed to measure IFNγ release (PE-IFNγ capture assay, Miltenyi) or cell surface CD69 and CD44 as detailed above.

### Statistical analysis

Tests of significance between pairs of data are reported as *p*-values, derived using a student's *t*-test with a two-tailed distribution and calculated at 95% confidence. Comparison of multiple sets of related data was achieved with one- or two-way analysis of variance (ANOVA). Survival curves compared using the log-rank (Mantel Cox) test. When present, error bars reflect standard error of measurement (SEM). Significance was set in each case to *p* < 0.05.

## SUPPLEMENTARY FIGURES


