# Supplementary material for: mTOR and MEK1/2 inhibition differentially modulate tumor growth and the immune microenvironment in syngeneic models of oral cavity cancer
Source: Oncotarget. 2015 Oct 22;6(34):36400–17. doi: 10.18632/oncotarget.5063 (PMC4742185; doi:10.18632/oncotarget.5063)
Supplement: Supplementary file 1 [file oncotarget-06-36400-s001.pdf]

SUPPLEMENTARY FIGURES

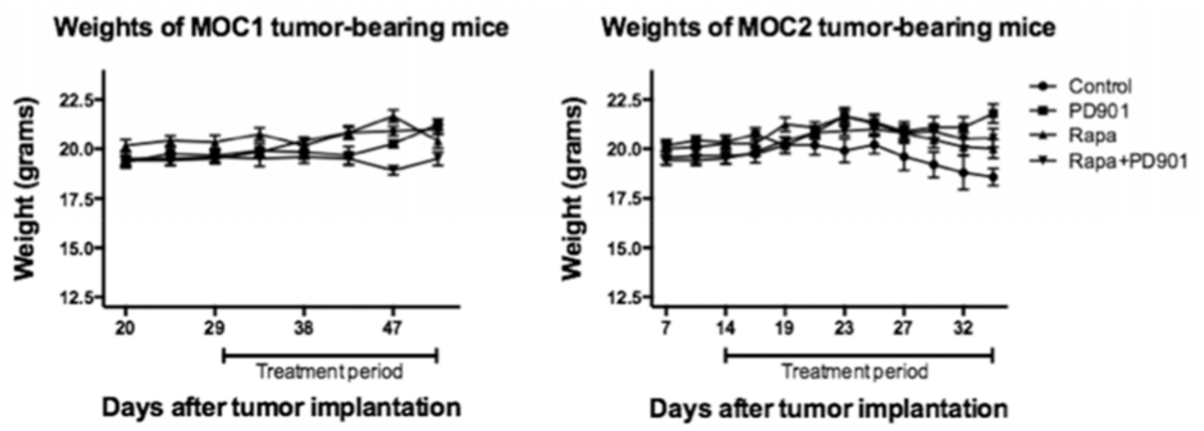

Supplementary Figure S1: Mouse weights during treatment with rapamycin and PD901. Mice were weighed at least twice weekly.

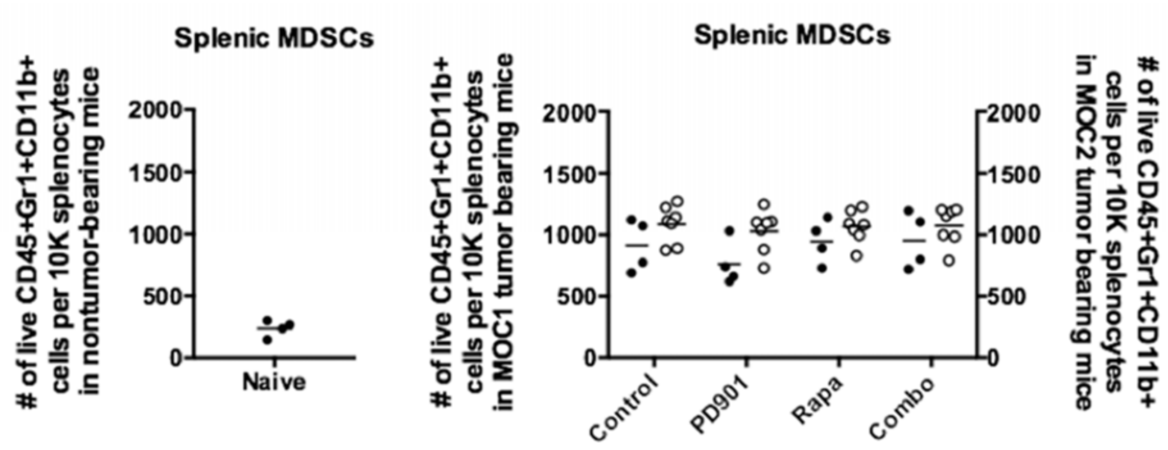

Supplementary Figure S2: Flow cytometric analysis of live CD45+Gr1+CD11b+ MDSCs in naïve (single axis plot) and MOC1 and MOC2 tumor-bearing mouse (double-axis plot) spleens. For the double-axis plot, left y-axis represents MOC1 mouse spleens and the right y-axis represents MOC2 mouse spleens. Dead cells excluded with 7AAD staining..

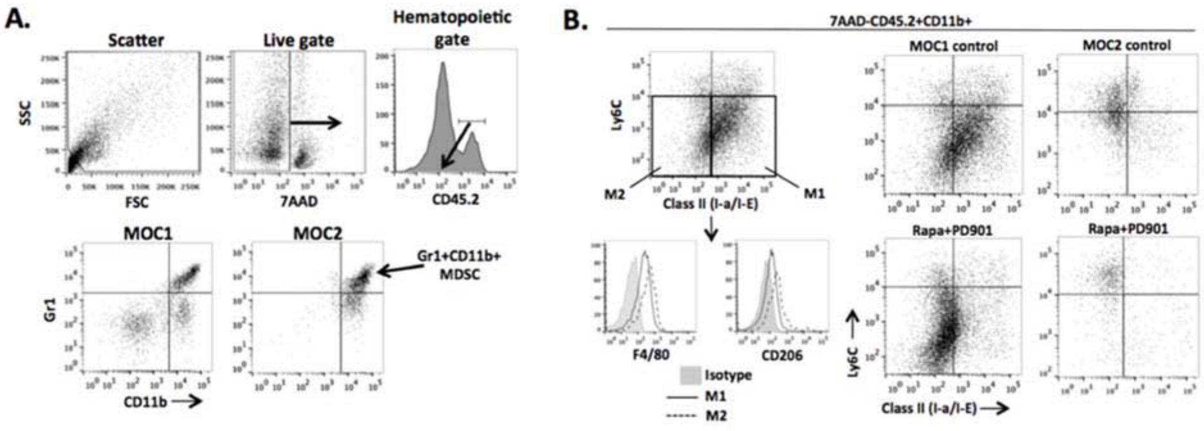

Supplementary Figure S3: Flow cytometry gating strategy for tumor infiltrating A. MDSCs and B. macrophages. Dead cells excluded with 7AAD staining.

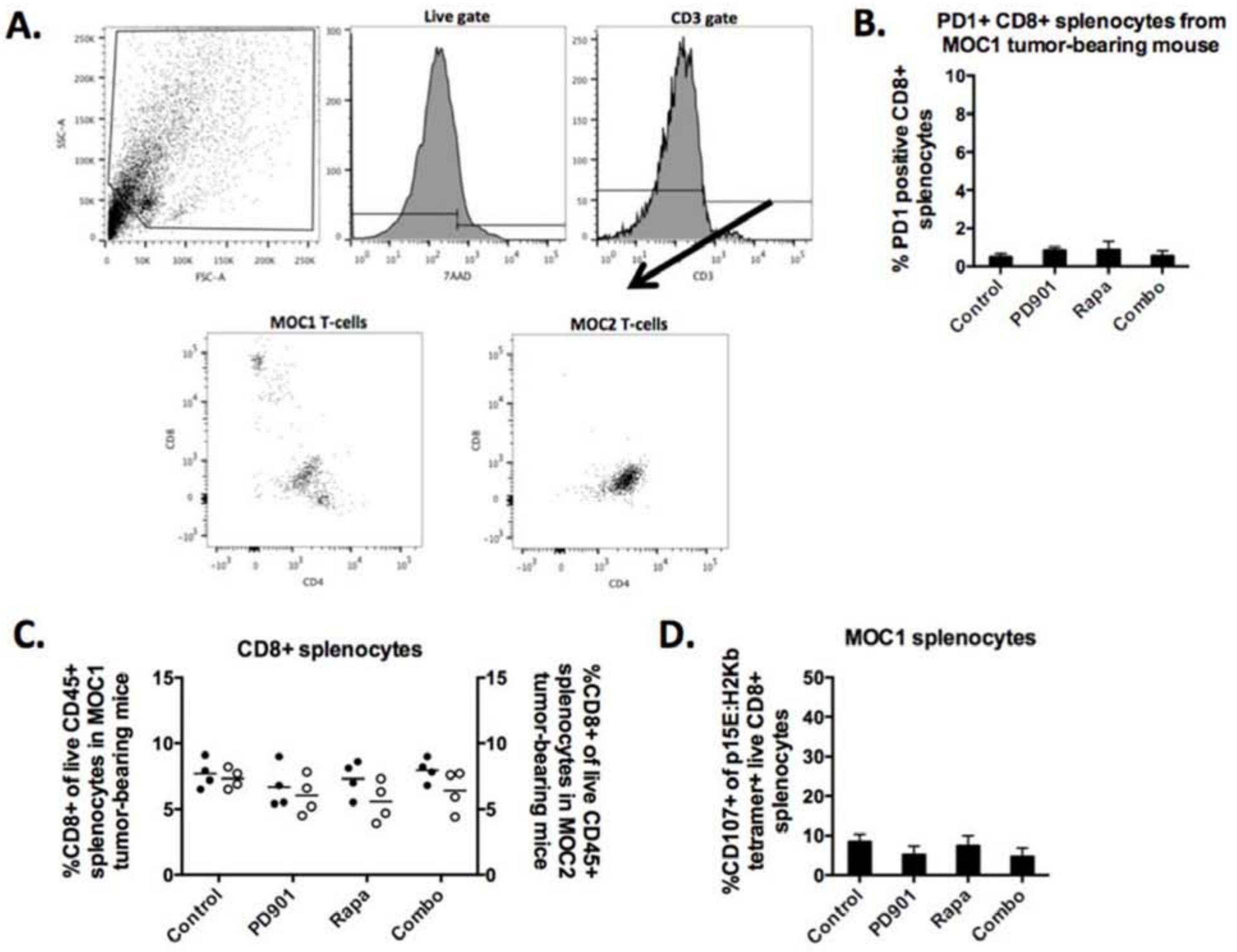

Supplementary Figure S4: A. Tumor infiltrating lymphocyte gating strategy. B. CD45+CD3+CD8+ splenocyte cell surface PD1 expression in treated MOC1 tumor-bearing mice. **C.** % of live, CD45+ splenocytes that are CD8+ in the spleens of MOC1 (left y-axis) and MOC2 (right y-axis) tumor-bearing mice. **D.** % of CD45+CD3+CD8+tetramer+ cells positive for cell surface CD107a expression in the spleens of MOC1 tumor-bearing mice.

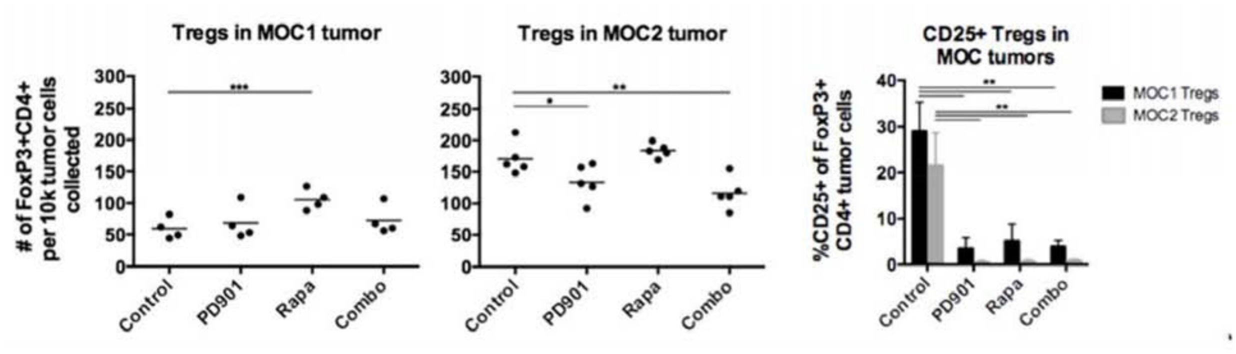

Supplementary Figure S5: Intracellular staining was performed to detect CD3+CD4+FoxP3+ Tregs. Number of MOC1 and MOC2 tumor-infiltrating Tregs shown, along with tumor-infiltrating Treg cell surface CD25 expression in treated tumors.

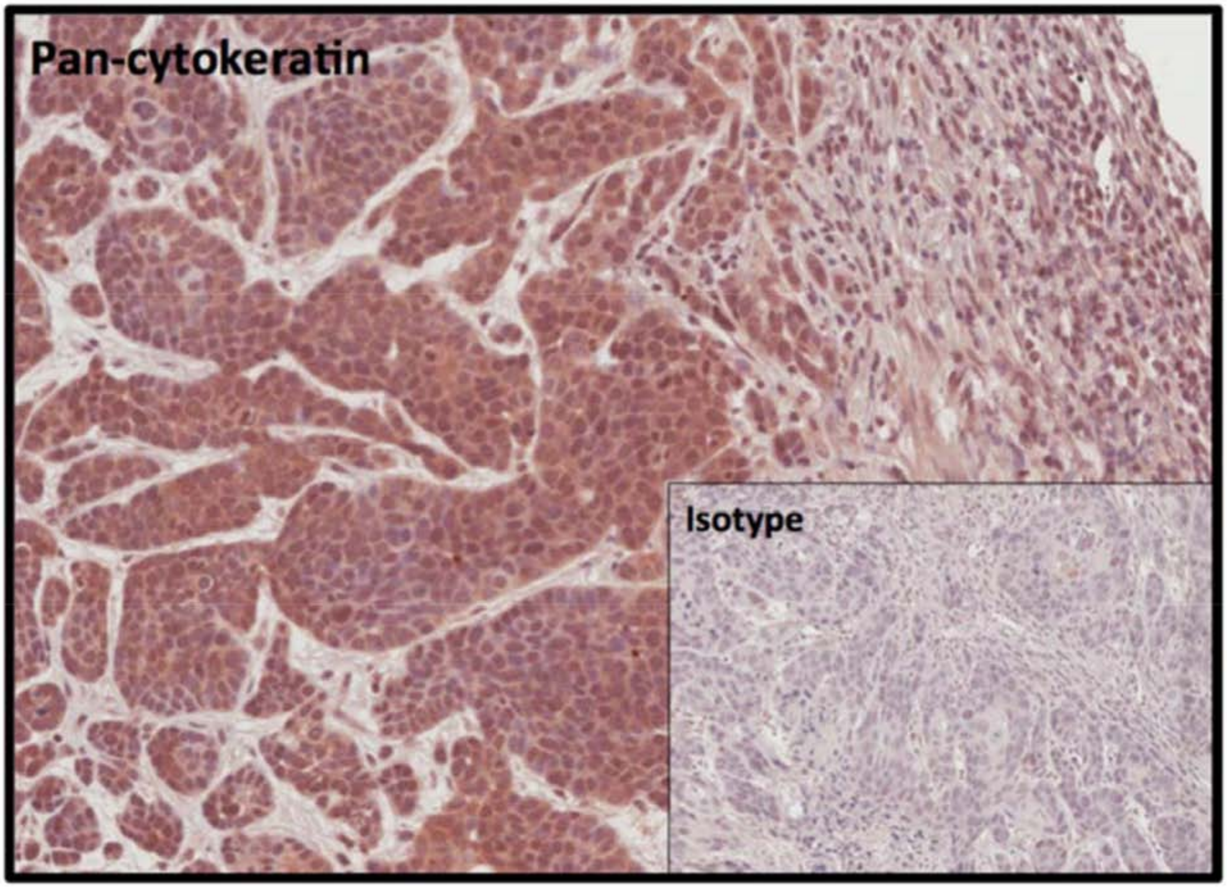

Supplementary Figure S6: Representative photomicrographs from pan-cytokeratin and isotype control (inset) immunohistochemistry. Pan-cytokeratin and isotype control stains were done for each treatment condition in MOC1 and MOC2 tumors to localize nests of tumor tissue and ensure specific antibody staining.
